# *Listeria monocytogenes* use multiple mechanisms to disseminate from the intestinal lamina propria to the mesenteric lymph nodes

**DOI:** 10.1128/spectrum.02595-24

**Published:** 2024-12-23

**Authors:** Joshua S. Nowacki, Grant S. Jones, Sarah E. F. D'Orazio

**Affiliations:** 1 Department of Microbiology, Immunology, and Molecular Genetics, University of Kentucky, Lexington, Kentucky, USA; Universidad Andres Bello, Santiago, Chile

**Keywords:** gastrointestinal infection, lymph nodes, foodborne, facutatively intracellular pathogens

## Abstract

*Listeria monocytogenes* are facultative intracellular bacterial pathogens that cause foodborne disease in humans. The bacteria can use the surface protein InlA to invade intestinal epithelial cells or transcytose across M cells in the gut, but it is not well understood how the bacteria traffic from the underlying lamina propria to the draining mesenteric lymph nodes (MLN). Previous studies indicated that *L. monocytogenes* associated with both monocytes and dendritic cells in the intestinal lamina propria. We show here that CCR2^−/−^ mice had a significant reduction in Ly6C^hi^ monocytes in the MLN but no change in bacterial burden following foodborne infection; thus, dissemination of *L. monocytogenes* associated with monocytes is not required for colonization of the MLN. To block CCR7-mediated trafficking of dendritic cells from the lamina propria, we treated mice with anti-VEGFR3 antibody (clone AFL4) prior to and during infection but did not see a change in dendritic numbers in the MLN as had been previously reported with other anti-VEGFR3-specific antibodies. However, increasing the number of circulating dendritic cells by treating mice with rFlt3L resulted in a significant increase in *L. monocytogenes* in the lymph nodes that drain the small intestine and the spleen. Whole-mount fluorescent microscopy of lymphatic vessels following ligated loop infection revealed both free-floating *L. monocytogenes* and cell-associated bacteria within lymphatic vessels. Together, these results suggest that *L. monocytogenes* can use multiple, redundant mechanisms to disseminate from the gut tissue to the MLN.

**IMPORTANCE:**

Consumption of the foodborne bacterial pathogen *Listeria monocytogenes* results in a wide spectrum of human disease from mild self-limiting gastroenteritis to life-threatening infections of the bloodstream, brain, and placenta. It is not well understood how the bacteria migrate from the intestines to the draining mesenteric lymph nodes, which are thought to serve as the last barrier to prevent systemic infections. Results presented here reveal multiple redundant mechanisms *L. monocytogenes* can use to disseminate from the ileum or colon to the mesenteric lymph nodes.

## INTRODUCTION

*Listeria monocytogenes* is a Gram positive facultative intracellular bacterium commonly found in the soil subsisting on decaying plant matter (1). *L. monocytogenes* is the causative agent of the foodborne illness listeriosis which typically occurs when ready-to-eat foods are contaminated during processing and consumed without being heated (2, 3). Ingested *L. monocytogenes* can invade the intestinal epithelium and, in some instances, use a direct route of spread to the liver via the portal vein, particularly when large inocula are used (4). However, it is thought that most of the orally acquired bacteria transit to the draining mesenteric lymph nodes (MLN) and escape into the bloodstream to cause systemic infection of the spleen and liver (5). Severe cases of *L. monocytogenes* infection have a high mortality rate (20%–30%), particularly in immunocompromised patients (6, 7).

It is thought that only a fraction of ingested *L. monocytogenes* survive passage through the harsh environment of the stomach, but exposure to low pH and bile results in transcriptional modifications that promote epithelial cell invasion (8–11). Invasion of the intestinal epithelium is still a relatively rare event even for these gut-adapted bacteria with most of the survivors being shed in feces (5, 12, 13). When InlA on the bacterial surface interacts with exposed E-cadherin on the basolateral surface of intestinal epithelial cells or goblet cells, a zipper-like mechanism of invasion is triggered that results in *L. monocytogenes* transcytosing directly across the cell into the underlying lamina propria (14–16). Alternatively, antigen-sampling M cells are also capable of phagocytosing *L. monocytogenes* and allowing for transcytosis across the intestinal epithelium into underlying Peyer’s Patches in the small intestine or lymphoid patches in the colon (17, 18).

The fate of *L. monocytogenes* after they cross the intestinal mucosa is not well understood. The predominant cell type found associated *with L. monocytogenes* in the underlying lamina propria is a Ly6C^hi^ monocyte (19). These cells develop in the bone marrow and stay sequestered there until the expression of the chemokine CCL2 is induced to direct the cells to infiltrate infectious foci in tissues (20, 21). Studies using an intravenous model of listeriosis suggest that monocytes aid in bacterial dissemination and invasion of the CNS following replication in the spleen and liver (22–24). Ly6C^hi^ monocytes isolated from the gastrointestinal tract, were resistant to invasion by *L. monocytogenes*, and did not serve as an intracellular growth niche (19, 25). Microscopic analysis of Ly6C^hi^ monocytes associated with *L. monocytogenes* indicated that the majority of the bacteria were found adhered to the monocyte surface (19). Dendritic cells (DC) are another migratory cell type present within the lamina propria which could transport *L. monocytogenes* to the MLN. Intestinal DC were found associated with *L. monocytogenes* in the lamina propria following oral infection (26). *Ex vivo* infection of DC isolated from the MLN of mice revealed that *L. monocytogenes* could invade primary conventional DC (cDC) and survive for several hours (26). Furthermore, mice lacking type 1 cDC (cDC1) have reduced bacterial burden in both the spleen and MLN (27–29) suggesting that dendritic cells play a key role in initial colonization of these tissues.

Colonization of the MLN is a critical step for systemic spread of *L. monocytogenes* (30), but it is currently unknown how the bacteria disseminate from the lamina propria to the MLN following foodborne transmission. One possibility is that *L. monocytogenes* attaches to or invades a migratory immune cell such as a Ly6C^hi^ monocyte or cDC and traffics to the MLN associated with that cell. Alternatively, it is possible that some free-floating *L. monocytogenes* could evade clearance by PMN present in the lymphatics and disseminate directly to the MLN. In this report, we tested each of these hypotheses and found evidence of both cell-free spread and spread via DC, suggesting *L. monocytogenes* likely use multiple pathways to disseminate to the MLN.

## RESULTS

### Mice deficient in C-C chemokine receptor type 2^−/−^ have no alterations in *L. monocytogenes* burden in the mesenteric lymph nodes

**Fig 1:**
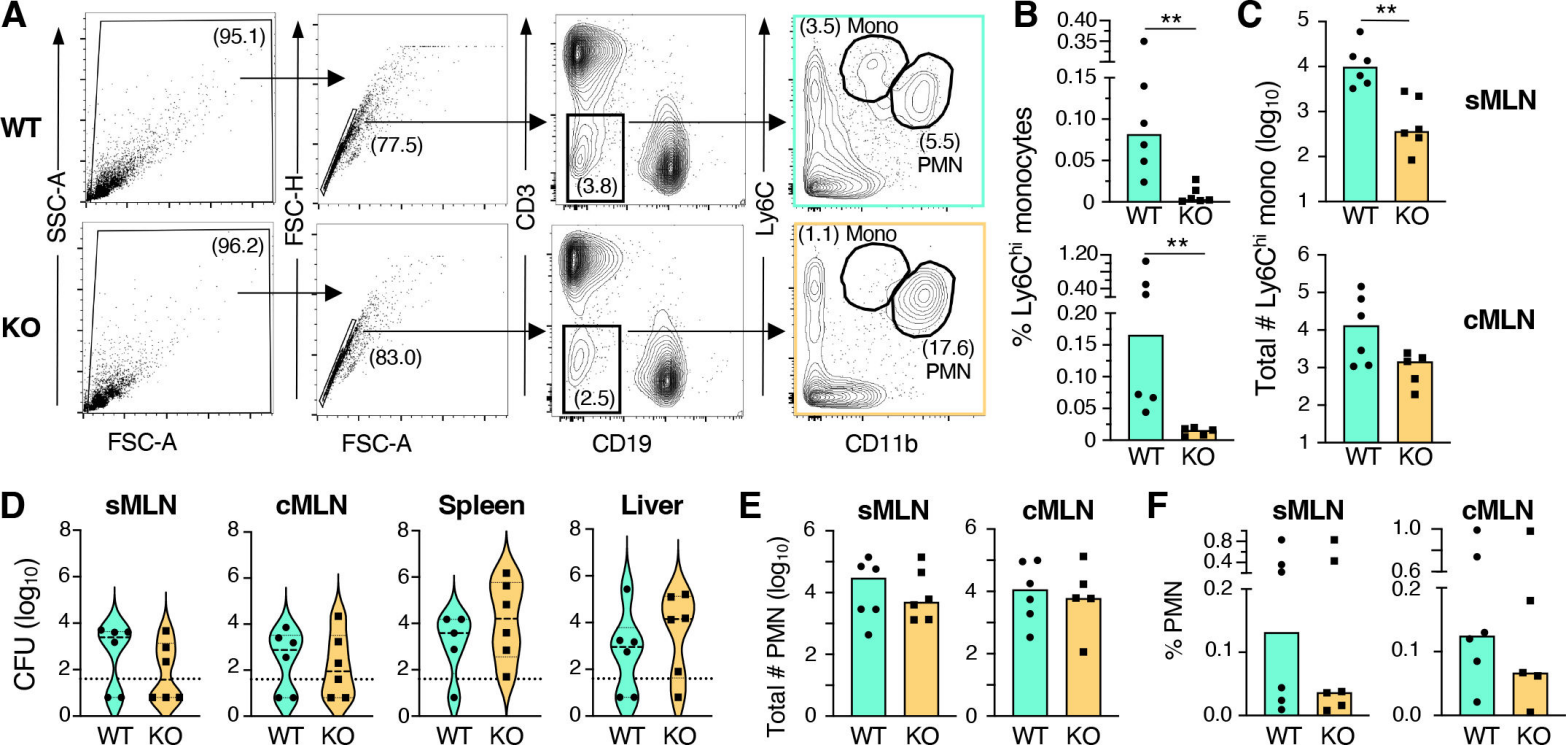
Inhibition of monocyte egress had no impact on *Listeria* burdens in the mesenteric lymph nodes. C57BL/6 (WT) and CCR2^−/−^ (KO) mice were fed 1.7–3.6 × 10^8^ CFU of *Lm* SD2001, and tissues were collected 3 days post infection. Pooled data for *n* = 6 mice analyzed in three independent experiments are shown. (**A**) Gating strategy used to identify Ly6C^hi^ monocytes (Mono) and neutrophils (PMN). Median percentage of live cells (**B**) and absolute number of monocytes (**C**) in MLN draining the small intestine (sMLN) and colon (cMLN). (**D**) Violin plots of the *Lm* SD2001 burden in the tissues 3 dpi; the dashed lines represent median, the dotted lines represent the limits of detection, and the vertical segments represent the interquartile range. Median number of PMN (**E**) and percentage of live cells (**F**) in both subsets of MLN as determined by flow cytometry. Significance was calculated for all panels using Mann-Whitney test for unpaired data (**P < 0.01).

We previously showed that Ly6C^hi^ monocytes were the most abundant cell population in the mesenteric lymph nodes that associated with *L. monocytogenes* at 2 and 3 days post infection (19). Although we did not find any evidence that *L. monocytogenes* replicated inside Ly6C^hi^ monocytes, small numbers of the bacteria were found attached to the cells (19). Since monocytes are migratory cells, we hypothesized that they might be able to transport *L. monocytogenes* from the intestinal lamina propria to the draining mesenteric lymph nodes (MLN). To test this, CCR2^−/−^ mice were used to alter the trafficking of Ly6C^hi^ monocytes in response to the chemokine CCL2. Monocytes in CCR2^−/−^ mice fail to exit the bone marrow efficiently during infection (31). CCR2^−/−^ and wild-type mice were infected with *L. monocytogenes* and 3 days post infection tissues were harvested for flow cytometry (Fig. 1A) and CFU quantification. The MLN were subdivided into nodes that drained the small intestines (sMLN), and the colon (cMLN) because previous studies have shown that *L. monocytogenes* colonize the colon to a much greater extent than the small intestine (12, 32).

As expected, CCR2^−/−^ mice had significantly fewer monocytes in the MLN (Fig. 1B), with the total number of cells being only a few hundred to a few thousand in the sMLN or cMLN of each mouse (Fig. 1C). However, there was not a significant difference in the bacterial burdens of the MLN in wild type or CCR2-deficient mice though approximately half of the wild type and CCR2-deficient mice had bacterial burdens below the limit of detection (40 CFU) (Fig. 1D). Since C57BL/6 mice are known to be relatively resistant to oral infection (12), we interpreted these data to mean that the infection had already been cleared in those mice. In the oral feeding model, systemic spread to the spleen and liver is thought to occur primarily from the MLN, and accordingly, we did not see significant differences in the number of bacteria in these tissues either (Fig. 1D). In agreement with these findings, the relative abundance of neutrophils in the sMLN and cMLN was similar in both wild type and CCR2^−/−^ mice (Fig. 1E and F).

Together, these results agreed with a previous experiment performed several years earlier in our lab in which CCR2^−/−^ and WT mice were infected with *L. monocytogenes*, and tissues were harvested 2 days post infection (Fig. S1A). In this earlier experiment, the MLN were not separated based on the intestinal tissue they drained, so each data point represents pooled MLN that were likely most representative of the sMLN. A similar reduction in monocytes was observed in the lymph nodes of CCR2-deficient mice compared to wild-type mice (Fig. S1B), and there was no significant difference in PMN number or percentage (Fig. S1C) in these animals. At this earlier time point, most of the mice still had quantifiable *L. monocytogenes* present in all tissue homogenates, but again, there was no significant difference in the bacterial burdens in the lymph nodes, spleen, or liver. Thus, efficient monocyte egress from the bone marrow was not required for dissemination of *L. monocytogenes* from the small or large intestine to the draining MLN.

### Vascular endothelial growth factor receptor 3 monoclonal antibody clone AFL4 does not inhibit C-C chemokine receptor type 7 expressing cells from migrating to the MLN

Another migratory cell type that can travel from the intestinal lamina propria to the lymph nodes is dendritic cells. We previously showed that *L. monocytogenes* did invade primary conventional dendritic cells isolated from the MLN (19, 26), so we hypothesized that these cells might promote the dissemination of intracellular bacteria. To test this, animals were treated with a VEGFR3-specific antibody prior to and after infection to inhibit dendritic cell transit to the lymph nodes. VEGFR3 is expressed on the surface of endothelial cells which line the lymphatic vessels that enter the MLN. Engagement of VEGFR3 by its cognate ligand VEGF induces the local production CCL21, a chemokine that attracts CCR7-expressing cells which includes both dendritic cells and T cells (33).

Previous studies showed that anti-VEGFR3 blocking antibody clone m4F-31C1 was capable of attenuating CCR7-mediated chemotaxis, resulting in reduced migration of T cells and dendritic cells to draining lymph nodes (33, 34). In one study, animals were injected intraperitoneally with antibodies every third day for 4 weeks, and another study injected clone m4F-31C1 directly into murine footpads and observed a subsequent reduction in CCL21 at local sites (33, 34). Together, these studies suggest that both short- and long-term antibody treatment can impact CCL21 production and subsequent immune cell recruitment. Clone m4F-31C1 antibody was developed by ImClone Systems, Inc. and is no longer commercially available, so clone AFL4 (purchased from BioLegend) was tested to determine if a similar effect on dendritic cell migration could be observed.

**Fig 2:**
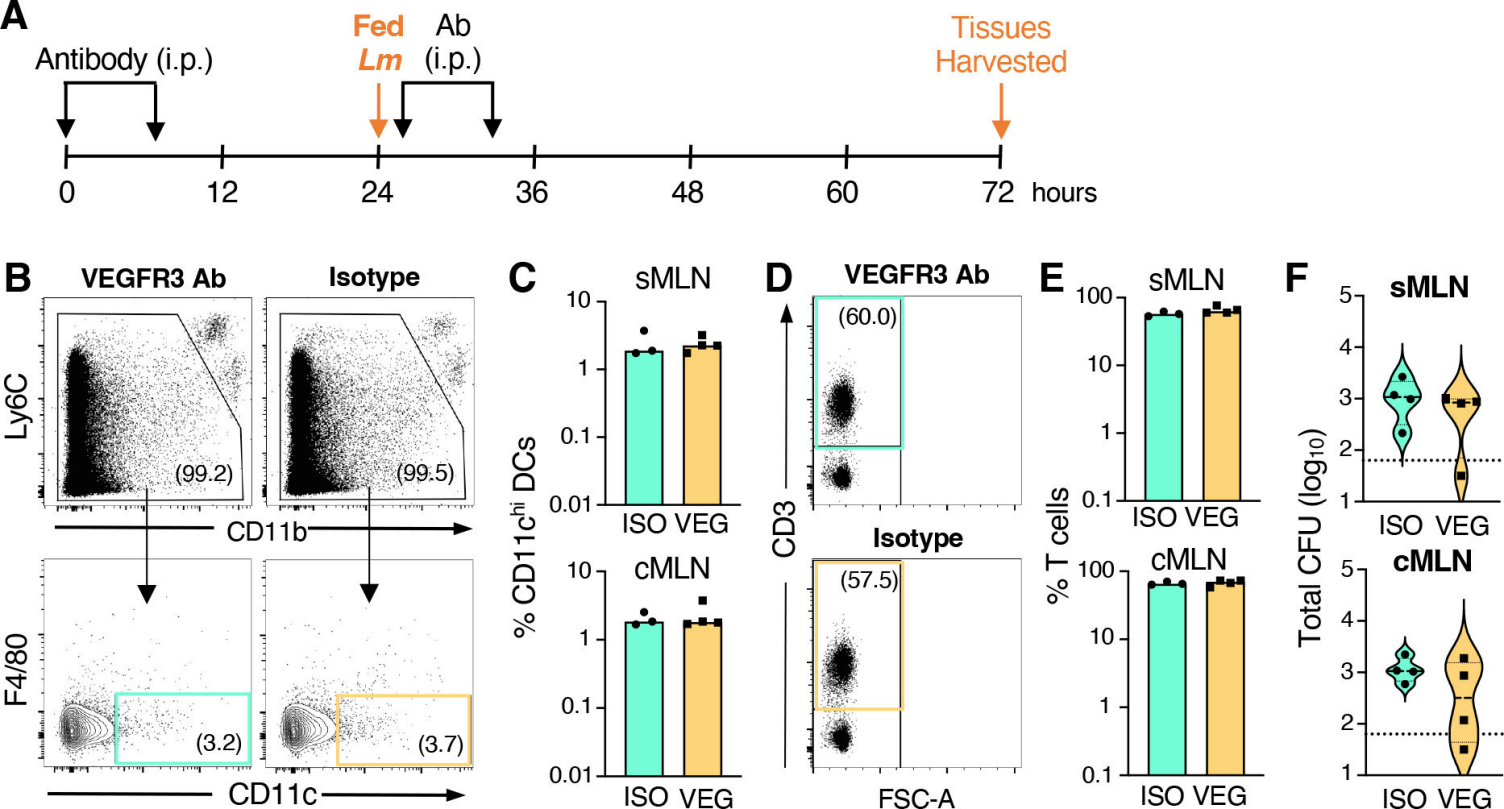
Treatment with anti-VEGFR3 clone AFL4 did not significantly alter CCR7^+^ cell recruitment to the MLN. Pooled data for *n* = 4 mice analyzed in four different experiments are shown. (**A**) BALB/cByJ mice were injected i.p. with 250 mg either anti-VEGFR3 (VEG) or isotype control (ISO) antibody and then fed 1–5 × 10^8^ CFU of *Lm* SD2000. Three additional i.p. injections of the same antibody occurred every 12 h after that and tissues were harvested at 72 hpi post infection for flow cytometry and CFU quantification. (**B**) Gating strategy used to define CD11c^+^ DC in MLN (pre-gated on live cells using FSC-A and SSC-A). (**C**) Median percentage of CD11c^+^ DCs in both subsets of MLN. (**D**) Gating strategy used to define CD3^+^ T cells in MLN (pre-gated on live cells). (**E**) Median percentage of T cells in both subsets of MLN. (**F**) Violin plots of the *Lm* SD2000 burden in the tissues 3 dpi; the dashed lines represent median, and the dotted lines represent limits of detection. Significance was assessed using a Mann-Whitney test for unpaired data.

Mice were given four intraperitoneal injections of clone AFL4 antibody, starting 24 h prior to infection, and tissues were harvested 3 days post infection (Fig. 2A). Flow cytometry was used to quantify the number of CD11c^+^ dendritic cells (Fig. 2B) or CD3^+^ T cells (Fig. 2D) in the MLN. No reduction in either the percentage (Fig. 2C and E) or the total number (data not shown) of CD11c^+^ and CD3^+^ cells in either the sMLN or the cMLN was observed following antibody treatment. Although a few animals treated with the clone AFL4 anti-VEGFR3 antibody had lower bacterial burdens in the MLN, the difference was not significant (Fig. 2F). Together, these data suggest that the anti-VEGFR3 clone AFL4 did not efficiently inhibit CCR7-mediated chemotaxis into the lymph node and that an alternate method needed to be used to assess the role of dendritic cells in *L. monocytogenes* dissemination.

### FMS-like tyrosine kinase 3 ligand-mediated DC expansion increases bacterial burden in the sMLN and spleen

If transit of intracellular bacteria was important for colonizing the MLN, we would expect increased migration of DC to the draining lymph nodes to generate greater *L. monocytogenes* burdens. Thus, instead of attempting to experimentally reduce DC migration, recombinant Flt3L was used to expand the DC population *in vivo* (35, 36). Use of Flt3L to induce dendritic cell differentiation *in vivo* is a well-established protocol that has been adapted for various areas of research (35–39). A standard protocol involves 10 µg injections administered daily over the course of nine days, typically resulting in a fourfold increase in the number of MHCII^+^CD11c^+^ cells within the lymph nodes, with some increases also detected by day 7 of this treatment schedule (36).

**Fig 3:**
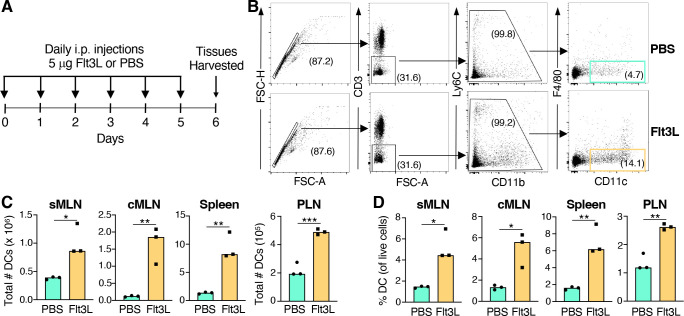
Six days of FLT3L injections were sufficient to increase the number and percentage of CD11c^+^ DC in the MLN fivefold. Pooled data for *n* = 3 mice per group analyzed in three independent experiments are shown. (**A**) Mice were injected (i.p.) with either phosphate-buffered saline (PBS) or 5 mg FMS-like tyrosine kinase 3 ligand (Flt3L) every day for 6 days and tissues were 24 h after the last injection. (**B**) Representative gating strategy for cells harvested from sMLN. Median number (**C**) and percentage (**D**) of CD11c^+^ DC as in lymphoid tissues including peripheral lymph nodes (PLN). Data from three independent experiments are shown. Significance calculated using a student’s *t* test for unpaired data (*P < 0.05; **P < 0.01; ***P < 0.001).

Pilot studies were performed to identify the minimal concentration and frequency of Flt3Linjections needed to observe a reproducible expansion of dendritic cells in uninfected animals. Animals were injected with either Flt3L or sterile saline (Fig. 3A), and tissues were harvested for flow cytometry (Fig. 3B). Daily injections of 5 µg rFlt3Lfor just 6 days resulted in a significant increase in both the total number and percentage of CD11c^+^ DC recovered from various lymphoid organs (Fig. 3C and D). Continuing rFlt3L treatment for 9 days did not further increase the number of dendritic cells produced (data not shown). Thus, an optimal expansion of dendritic cells could be achieved using half of the standard dose for less than a week.

**Fig 4:**
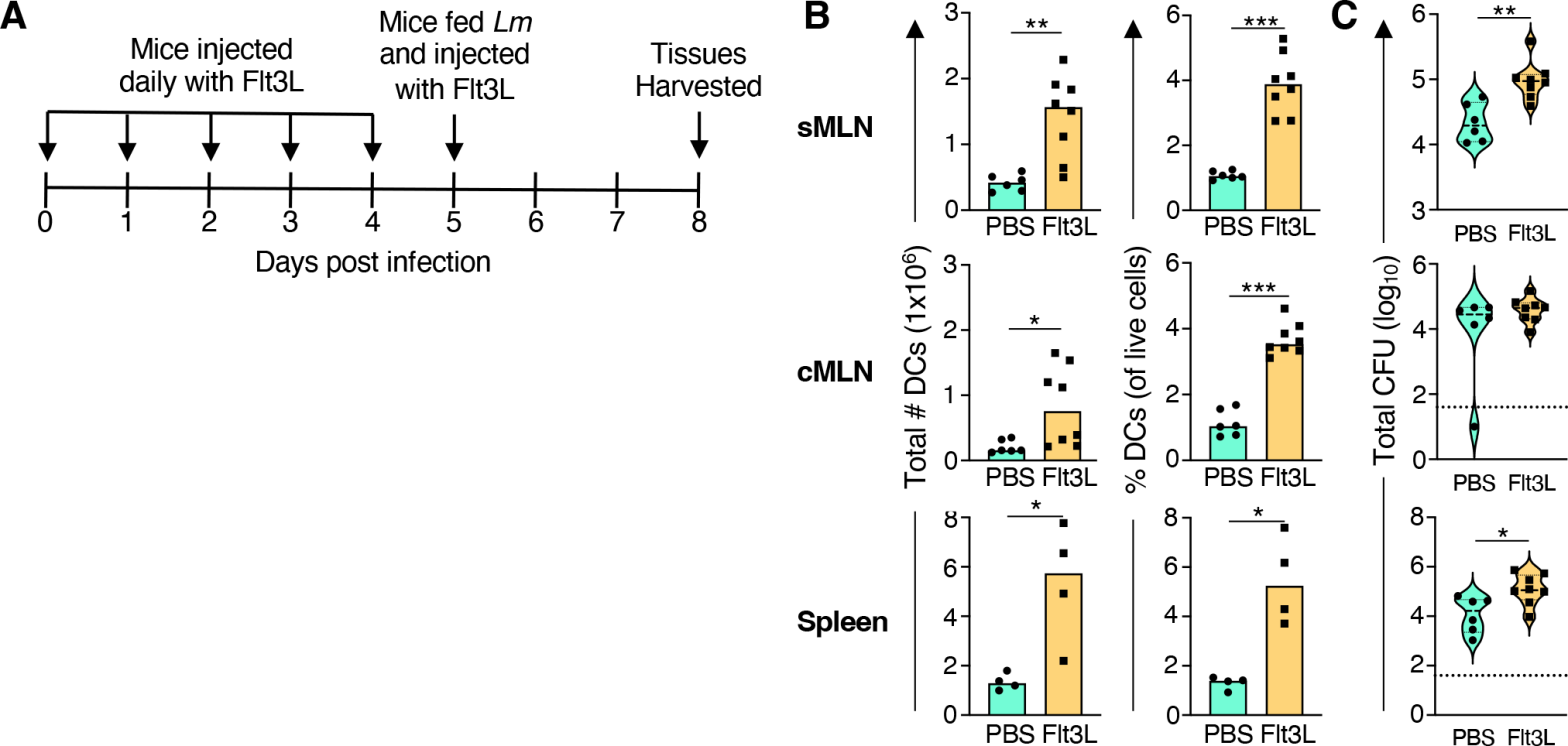
Increasing the number of dendritic cells resulted in increased bacterial burdens in the sMLN and spleen. (**A**) BALBc/ByJ mice were injected with either murine Flt3L or PBS for 6 days to expand DC differentiation and then infected with 2–8 × 10^8^ CFU of *Lm* SD2000. Tissues were collected 3 days post infection. Four independent experiments with *n* = 2 mice per group were performed. (**B**) Median number and percentage of live cells for CD11c^+^ DC in sMLN, cMLN, and spleen; CD11c^+^ DC were identified using the gating strategy shown in Fig. 3 only for experiments #3 and #4. (**C**) Violin plots of the *Lm* SD2000 burden in tissues; dashed lines represent medians, and the vertical segments represent the interquartile range. Dotted lines across the graphs represent limits of detection. Significance was calculated using Mann-Whitney test for unpaired data (*P < 0.05; **P < 0.01; ***P < 0.001).

Using the rFlt3L treatment described above, mice were infected with *L. monocytogenes* immediately following the last injection of rFlt3L (or saline), and the MLN and spleen were harvested 3 days post-infection (Fig. 4A). Flow cytometric analysis confirmed a significant increase in both the number and the percentage of CD11c^+^ dendritic cells in all three tissues (Fig. 4B). In the sMLN, a fourfold increase in the number of CD11c^+^ dendritic cells was observed, which correlated with a fivefold increase in bacterial burden in the treated animals (Fig. 4C). Likewise, the number of CD11c^+^ dendritic cells increased fourfold in the spleen, and a sevenfold increase in bacterial burden was noted (Fig. 4C). We also observed a fourfold increase in *L. monocytogenes* in the liver (data not shown), but we did not measure dendritic cell numbers in the liver. No significant difference in bacterial burdens was observed in the cMLN despite a fourfold increase in the number of dendritic cells (Fig. 4C). These results indicated that Flt3L-mediated expansion of CD11c^+^ dendritic cells prior to infection reproducibly increased bacterial burdens in the lymph nodes that drained the small intestine as well as systemic spread to the spleen.

### *Listeria monocytogenes* is found both associated with cells and free floating in murine lymphatic vessels using a ligated loop model

*Lm* are facultative intracellular pathogens which can exist both intracellularly and extracellularly within a host (32). Although PMN can clear a large portion of the extracellular bacteria, it is possible some *L. monocytogenes* could avoid phagocytosis and travel through lymphatic vessels without being associated with a migratory cell (30, 32). To assess this, a ligated ileal loop model was used to allow for direct control of the amount of *L. monocytogenes* and the timing of entering the ileum. *Lm* were injected directly into a sutured section of the ileum, and bacterial dissemination was examined 45 min later by confocal microscopy of whole-mount mesentery.

**Fig 5:**
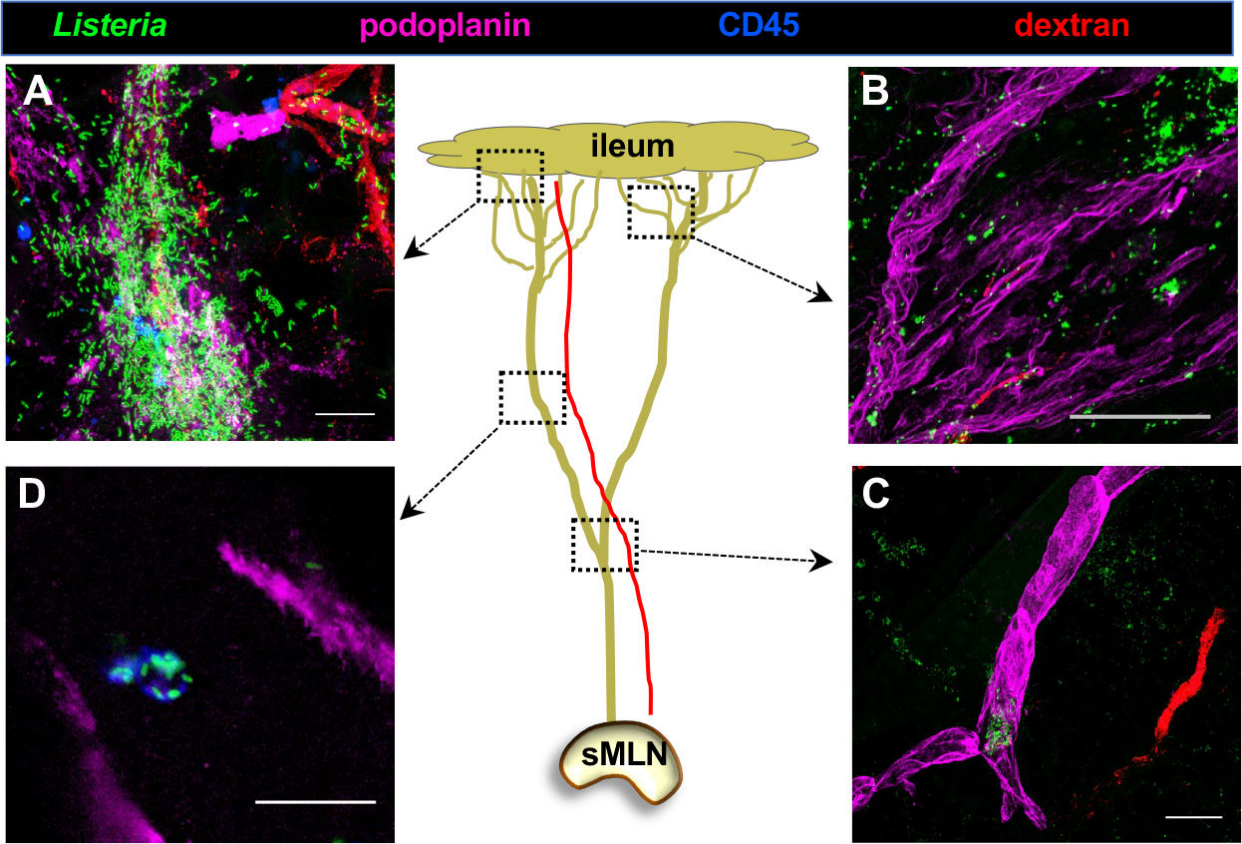
Both cell-associated and free-floating *L. monocytogenes* were observed in mesentery lymphatic vessels during ligated loop infection. BALBc/ByJ mouse ileal loop was injected with 10^9^ CFU GFP^+^ *Listeria,* and tissues were harvested 45 min post infection. Just prior to euthanasia, fluorescently conjugated dextran was injected intravenously. The center cartoon depicts a single plexus leading from the ileum to the draining sMLN; the dotted lines indicate the areas analyzed in the surrounding representative images. (**A**) Hundreds of *L. monocytogenes* are found proximal to the intestinal lamina propria. (**B**) A network of lymphatic vessels near the gut tissue. (**C**) Large collecting vessel more distal to the gut with a cluster of *L. monocytogenes* at the branch point. A blood vessel containing dextran is seen on the right. (**D**) A CD45+ cell with several attached *Lm* migrates through the vessel. Representative images from one of two independent experiments are shown; all images are Z stacks except (**D**) which shows a single *z* plane. Scale bars, 20 mM.

Representative images depicting *Lm* visualized from different locations within the mesentery are shown in Fig. 5. Hundreds of bacteria were visible close to the intestinal lamina propria, and they appeared to have transited out of the lymphatic vessel and into the surrounding adipose tissue (Fig. 5A). Further from the ileum, there were fewer bacteria, but they also appeared to be outside of the lymphatic (podoplanin^+^) vessels (Fig. 5B). The relative frequency of *L. monocytogenes* decreased with distance from the ileum, as shown in the representative image from a distal portion of the lymphatic vessel in Fig. 5C. At this location relatively closer to the sMLN, clusters of *L. monocytogenes* were visualized within the lymphatic vessels, but none were observed in the nearby blood vessel (dextran^+^). In addition to extracellular bacteria, we also observed a small number of *L. monocytogenes* co-localizing with CD45^+^ cells (Fig. 5D). These results suggest that *L. monocytogenes*, when present in the ileum, are capable of entering the draining lymphatic system using both cell-associated and free-floating mechanisms of dissemination.

## DISCUSSION

**Fig 6:**
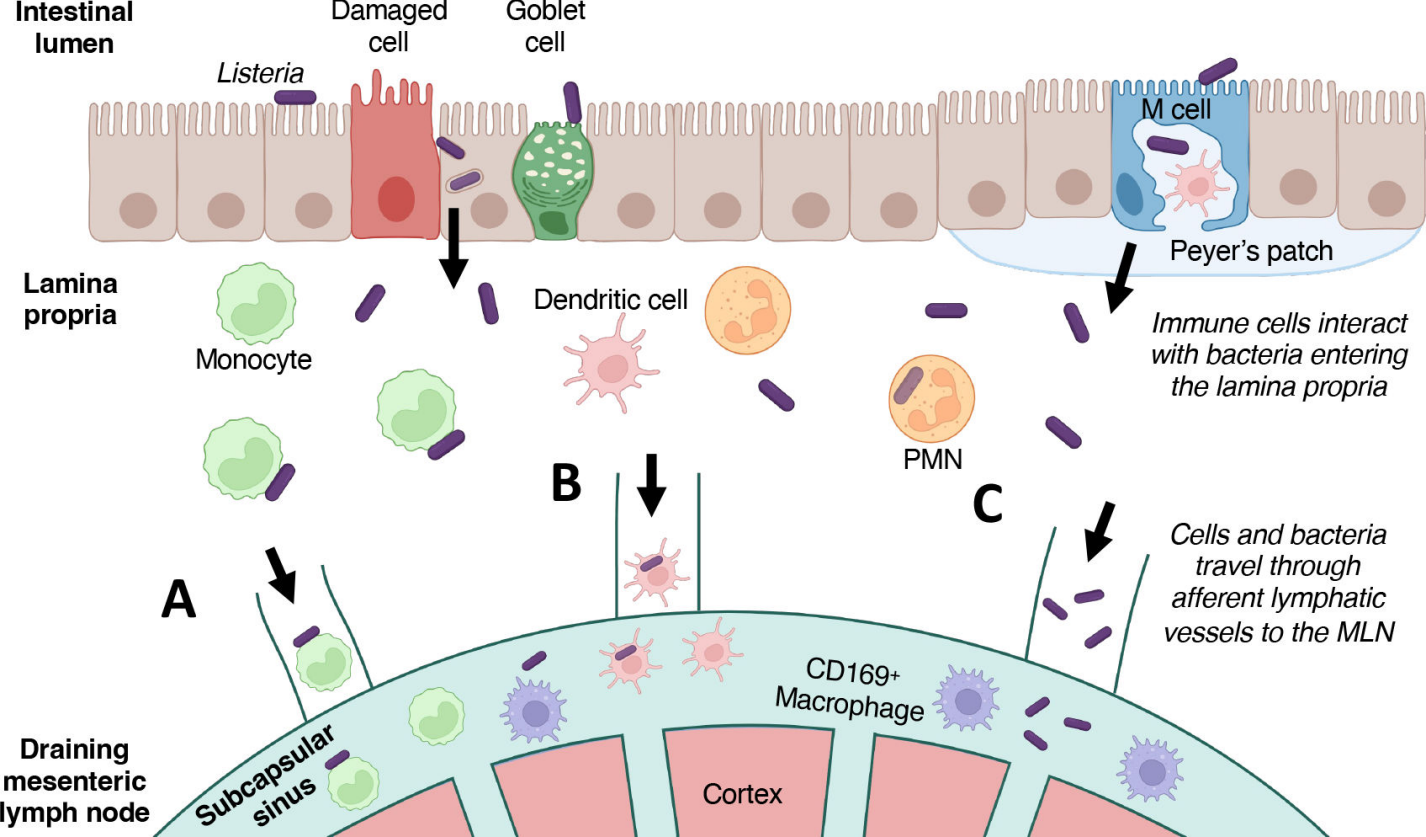
Proposed model of dissemination from intestinal lamina propria to the MLN. Ingested *L. monocytogenes* (purple) in the gut lumen can cross the mucosal barrier by either InlA-mediated uptake into epithelial cells or transcytosis via M cells. Once in the underlying lamina propria, *Lm* can interact with a variety of immune cells, including activated PMN and macrophages which promote clearance of extracellular bacteria. (**A**) Ly6C^hi^ inflammatory monocytes do not readily phagocytose *Listeria* but may transport surface-associated bacteria to the MLN. (**B**) Migratory CD11c^hi^ CD103^+^ cDC can internalize *L. monocytogenes* and then migrate to draining lymph nodes to present antigen to T cells. Previous data suggest that *Listeria* survive for several hours within DC. (**C**) Extracellular bacteria that are not cleared by phagocytes can disseminated free floating through the afferent lymphatic vessels to enter the subcapsular sinus of the lymph node. Together, these three mechanisms make up our proposed routes of dissemination for *Lm* to travel from the LP to the MLN.

Invasion of the intestinal epithelium by *L. monocytogenes* has been studied in detail using both *in vitro* and *in vivo* approaches, but how the bacteria interact with innate immune cells in the underlying lamina propria is still not well understood (40). Based on the results presented here, our working model for *L. monocytogenes* dissemination to the draining MLN is shown in Fig. 6. Inflammatory monocytes are the most abundant cell type in the intestinal lamina propria that associate with *L. monocytogenes* (19), and these cells may transport the bacteria to the MLN, but they are not required for colonization of the lymph nodes (Fig. 6A). Dendritic cells readily internalize *L. monocytogenes* (26), and we showed here that increasing cDC1 output resulted in increased bacterial burdens in the MLN, suggesting that these migratory cells can also transport *L. monocytogenes* (Fig. 6B). However, even DC are not likely to be required for transport since we found evidence that free floating *L. monocytogenes* can also transit through the lymphatic vessels to reach the MLN (Fig. 6C). These three dissemination pathways are likely to be redundant; as a facultative intracellular pathogen, *L. monocytogenes* can use one or more of these mechanisms to disseminate to the MLN (Fig. 6).

The initial observation that motivated this work was that the intracellular replication of *L. monocytogenes* was required to efficiently colonize the MLN (32). At the time, it was not known if intracellular replication was needed to disseminate from the intestinal lamina propria to the MLN or for subsequent survival after bacteria reached the lymph nodes. However, we recently showed that MLN do not have sufficient exogenous lipoate to promote extracellular growth of *L. monocytogenes* (30). In combination with the data presented here, this supports a model in which *L. monocytogenes* can use any of the three pathways shown in Fig. 6 to reach the draining MLN without needing to replicate intracellularly. Once inside the lymph node, exponential expansion of *L. monocytogenes* is possible only by invading a permissive cell type and replicating in the cytosol. Free-floating bacteria that enter the lymph node are expected to be engulfed by CD169^+^ macrophages in the subcapsular sinus. In the i.v. model of listeriosis, CD169^+^ marginal zone macrophages in the spleen rapidly filter *L. monocytogenes* from the blood and are thought to quickly restrict intracellular growth of the bacteria (41, 42). Bacteria associated with either monocytes or dendritic cells could be transported deeper into the node to the paracortex region, but neither of these cell types serves as a replicative niche for *L. monocytogenes* (19, 26). Tucker et al. recently showed that stromal cells in the MLN can support intracellular growth of *L. monocytogenes* (43), but the mechanism by which bacteria trapped in phagocytes access these permissive cells is still not clear.

Mice that lack cDC1 have significantly reduced colonization of the spleen following i.v. inoculation and a smaller defect in the MLN following oral transmission (27–29). In the systemic model, multi-photon intravital imaging was used to visualize host pathogen interactions in the marginal zone of the spleen, and within minutes, *L. monocytogenes* were seen co-localized with CD11c^+^ cells extending dendrites toward the bacteria (44). Over the course of the next 24 h, the dendritic cells began to move toward the T cell zone of the spleen (45, 46), and it is thought that these cells transport *L. monocytogenes* to this region where further replication occurs. Multiple studies have suggested that cDC1 serve as an intermediate cell type, which can be “trans-infected” by macrophages that have taken up *L. monocytogenes* (42, 47), but this mechanism of “trans-infection” has not been elucidated. Other DC subsets do not appear to have any effect on bacterial burdens in the MLN. For example, IRF4^−/−^ mice which lack type 2 cDC (cDC2) had no difference in bacterial burdens in the MLN following *L. monocytogenes* infection (28). Plasmacytoid DC (pDC) were found to be resistant to *L. monocytogenes* infection, but it is possible *L. monocytogenes* may adhere to the cellular surface (48).

In this study, Flt3L-mediated DC expansion altered *L. monocytogenes* colonization of the lymph nodes that drained the small intestine, but not the colon. We previously demonstrated that both colonic epithelial cells and the underlying lamina propria had higher CFU than those of the ileum (12); therefore, it is possible that larger bacterial burdens can overwhelm innate immune clearance mechanisms in the colon causing a higher proportion of free-floating bacteria to spread from the colon to the cMLN. However, invasion of the mucosal barrier of the small intestine was much more dependent on InlA than invasion of the colonic epithelium (12). This suggests that the mechanism used by the bacteria to cross the intestinal epithelium may influence the pathway used to disseminate to the MLN. Alternatively, it is also possible that there are differences in the trafficking patterns or functions of cDC in the sMLN and cMLN. Houston et al. found no apparent difference in the proportion of cDC1 or cDC2 subsets in the sMLN or cMLN, but the colon-draining nodes do have more CX3CR1^+^ cells (49). This is significant as CX3CR1 is a chemokine receptor which is important for dendritic cell development as well as bacterial and viral clearance (50–52).

CCR2-deficient mice have a significant decrease in the migration of monocytes from the bone marrow to sites of infection, but as shown here and in other reports, Ly6C^hi^ monocytes are still present in the MLN at approximately 10% of the level seen in wild-type animals. It is possible that this residual population is sufficient to transport *L. monocytogenes* from the intestinal lamina propria. Alternatively, monocytes are known to produce cytokines such as TNF-α and IL-1, which promote bacterial clearance (53, 54), and therefore, any reduction in bacterial burden that may been due to decreased transport could have been offset by increased survival of the bacteria due to a diminished pro-inflammatory response. Monocytes have also been implicated to transport *L. monocytogenes* across the blood brain barrier (22–24), and this is presumed to involve intracellular bacteria. Our own previous work suggests that monocytes are not a replicative niche for *L. monocytogenes* (19), but the discrepancy in these published studies may be due to phenotypic differences in particular subsets of monocytes that are not yet well defined.

Using a high dose ligated loop model, both cell-associated and free-floating *L. monocytogenes* were observed within lymphatic vessels that drain the small intestine. Lymphatic vessels are more permeable than blood capillaries and are designed to bring fluid present in tissues to the filtering lymph nodes, which act as a final barrier to accessing the bloodstream and systemic spread of infection (55). *L. monocytogenes* could use flagella to move through lymphatic vessels although most strains rapidly downregulate flagellum expression *in vivo* (56–59). Bacterial motility is presumably not required to traffic within lymphatic vessels because a recent study showed that nonmotile *Yersinia pestis* spread via the lymphatics to skin-draining lymph nodes (60). Lymphatic vessel endothelial receptor-1 (LVYE-1) is highly expressed on the inner surface of lymphatic vessels and can bind to hyaluronic acid capsules of bacteria. This interaction has been implicated in the lymphatic trafficking of *Streptococcus pyogenes* (61).

Our microscopic analysis revealed *L. monocytogenes* in the mesentery outside of both the blood and lymphatic vasculature. Kuan et al. showed that the inherent permeability of collecting lymphatic vessels enabled the distribution of lymph and lymph components into the surrounding fat tissue (62). We presume that infection with such an overwhelming dose of bacteria in a static ligated loop can easily result in significant spillage of *L. monocytogenes* into the fat pads of the mesentery, but it is not known how physiologically relevant this localization pattern is for natural foodborne transmission in humans. Adipose tissue can be readily colonized by *L. monocytogenes* following subcutaneous injection of fat pads, and this results in an infiltration of immune cells within the perinodal fat tissue and subsequent colonization of the draining lymph nodes (63). Thus, it is possible that dendritic cells could internalize *L. monocytogenes* in the perinodal fat tissue and then transport the bacteria to the MLN, but this may occur only in situations of high bacterial burden, because it has been shown that adipocytes and iNOS-producing cells in fat tissues are effective at clearing bacterial infections (63, 64). Colonization of adipose tissue has not been well studied in either the intravenous or oral mouse models of listeriosis and represents an important area of future study.

## MATERIALS AND METHODS

### Bacteria

Mouse-adapted (InlA^m^ -expressing) *L. monocytogenes* EGDe derivatives SD2000, SD2710 (constitutive GFP), and SD2001 (GFP^neg^ vector control) were previously described (32). All strains were cultured using Brain Heart Infusion (BHI) broth at 30°C to early stationary phase, aliquoted, and stored at −80°C prior to use (65).

### Mice

Female BALBc/ByJ mice (stock # 001026), B6.129S-4Ccr2 mice (CCR2^−/−^ stock # 004999), and C57BL/6J mice (stock # 000664) were obtained from The Jackson Laboratory (Bar Harbor, ME). All BALBc/ByJ mice were adapted to a 14 h light cycle (7 p.m. to 9 a.m.) for at least 2 weeks prior to use in experiments. Mice were between 6 and 9 weeks of age at the start of each experiment, and all procedures were approved by the Institutional Animal Care and Use Committee (IACUC) at the University of Kentucky.

### Foodborne infection of mice

Mice were infected by the natural route of transmission as described previously (66, 67). Briefly, mice were placed in cages with raised wire floors to prevent coprophagy, and food was withheld for 16–24 h prior to infection. Aliquots of *L. monocytogenes* were thawed in BHI broth for 1.5 h at 30°C, washed once in phosphate-buffered saline (PBS; Life Technologies cat no. 14190-144), added to a 3 mm piece of white bread (Kroger), followed by addition of melted salted butter (Kroger). Mice were fed the contaminated bread at the beginning of their dark cycle, and chow was returned immediately after infection. The inoculating dose was confirmed by titering the number of bacteria present on 1–2 leftover bread pieces by preparing serial dilutions in sterile water and plating on BHI agar.

### Lymph node cell isolation

Mesenteric lymph nodes that drained the small intestine (sMLN) and colon (cMLN) were aseptically harvested as described previously (49). Peripheral lymph nodes (PLN) consisted of axial plus inguinal nodes. All lymph nodes were sectioned into quadrants with a sterile scalpel blade and then incubated shaking (250 rpm) at 37°C for 30 min in a 50 mL conical tube containing a sterile 2 cm stir bar and 4 mL of a solution consisting of Collagenase IV (300 U/mL; Worthington), DNase I (120 U/mL; Worthington), 20 µM HEPES, and 5% fetal bovine serum (FBS; GeminiBio cat # 100-106) in RPMI1640 (Life Technologies cat no. 21870-084). Following collagenase digestion, one-tenth of the solution was used for CFU quantification, and the remainder was passed through a nylon cell strainer (40 µm pore size; VWR) to obtain a single cell suspension. Cell density and viability were assessed by trypan blue staining and manual counting in a hemacytometer.

### Flow cytometry

Single-cell suspensions were incubated with fluorescently conjugated monoclonal antibodies specific for the following murine cell surface markers: F4/80 (clone BM8) and CD117 (clone 2B8) from eBiosciences; CD3 (clone 17A2), Ly6G (clone 1A8), Ly6C (clone HK1.4), CD11c (clone N418), CD11b (clone M1/70), CD103 (clone 2E7), CCR7 (clone 4B12), CD16/32 (Fc Block; clone 93), and CD19 (clone 6D5) from BioLegend. Following incubation, cells were fixed with 10% neutral buffered formalin (VWR cat no. 16004-128) prior to analysis. Flow cytometry data were obtained with an LSRII cytometer (BD Biosciences) and analyzed with FlowJo software (version 10).

### Quantification of bacterial loads in tissue homogenates

Livers and spleens were harvested aseptically in 2.5 mL (spleens) or 4 mL (liver) of sterile water and homogenized at power level 4 for 30 s using a VWR 200 tissue homogenizer. Serial dilutions of tissue homogenates or lymph node solutions were plated on BHI agar (Difco) and incubated at 37°C. CFU were counted the following day and reported as total CFU per tissue.

### Treatments to alter dendritic cell number and trafficking

To alter DC trafficking, mice were given two intraperitoneal injections (125 µg) 5–8 h apart with either monoclonal antibody specific for murine Vascular Endothelial Growth Factor 3 (anti-VEGFR3; clone AFL4 purchased from BioLegend) or the isotype control Ultra LEAF Purified Rat IgG2κ (clone RTK2758 purchased from Biolegend) on day −1. On day 0, mice were infected and given two more intraperitoneal injections (125 µg) 5–8 h apart. To increase output of DC, mice were given daily i.p. injections of FMS-like Tyrosine Kinase 3 Ligand Flt3L (5 µg murine carrier free; Biolegend).

### Splenocyte isolation

Spleens were aseptically harvested into 60 mm dishes and injected with 1 mL of 100 U/mL collagenase D (Roche) using a 22G needle. Spleens were then torn into pieces using the needle and sterile forceps. Spleen fragments were transferred to a 10 mL centrifuge tube, the dish was washed with 2 mL of 100 U/mL collagenase D solution, and the collected cells were added to the same tube. Following the addition of 1 mL of 400 U/mL collagenase D, the tube was incubated for 60 min at 37°C/7% CO_2._ The digested spleen fragments were then pushed through a mesh screen using the plunger of a 3 mL syringe, and the screen was washed with 4 mL of 100 U/mL collagenase solution. The single-cell suspension was treated with ACK lysis buffer (150 mM NH_4_Cl, 10 mM KHCO_3_, and 0.1 mM Na_2_EDTA) to lyse red blood cells.

### Ligated loop infection model

Food was withheld from mice housed on raised wire floors for 12 h to promote gastric emptying. Just prior to surgery, mice were anesthetized using 3%–5% isoflurane and moved to a recirculating water heating pad in a BSL-2 safety cabinet to maintain body temperature. A small incision was made at the midline, both cecum and ileum were exteriorized, and a 4 cm section of ileum was sutured at both distal and proximal ends using circumferential sutures that allowed for circulation of blood and lymphatic flow. Following sutures, 10^9^ CFU of *L. monocytogenes* SD2710 suspended in 100–150 μL of sterile PBS was injected directly into the proximal end of the ileal loop using a 27G needle, and then the intestines were reinserted into the body cavity. Following suture, mice were maintained on isoflurane using a nose cone and were monitored for 45 min. Two minutes prior to euthanasia, mice were injected with 400 mg Texas-Red dextran (Dextran, Texas Red, 70,000 MW, Lysine Fixable, Molecular Probes, cat# D1864) into the facial vein. The GI tract was then aseptically harvested.

### Whole mount confocal microscopy

The intact small intestines and attached mesentery were fixed in freshly made 4% paraformaldehyde in PBS (pH 7.4) for 24 h. Then, the mesenteric vessels were removed from gut, washed with PBS, and permeabilized by treating with 1% Triton-X100 in PBS for 4 h with agitation. A solution consisting of 1% BSA, 0.2% Triton-X100 in PBS was added, and the tissues were allowed to sit at room temperature for 2 h. Directly-conjugated antibodies were added at 1 µg/mL (eBioscience CD45/eFluor450 clone 30-F11 & eBioscience Podoplanin/eFluor660 clone eBlo8.1.1) and incubated overnight at 4°C, and then the tissue was washed with PBS and transferred to a m-Slide 2 well glass-bottom dish (ibidi #80287) with 50–100 μL anti-fade non-hardening liquid mounting media (Vectashield) and a coverslip was added. Slides were imaged using a laser scanning inverted confocal microscope (Leica TCS SP5). Sequential scans at 400 Hz were performed using a 405 diode (CD45), Argon (GFP), HeNe 594 (Texas Red), and HeNe 633 (podoplanin) laser to excite the fluorophores. A 40× or 63× oil objective was used to image along the *z* axis where 30–60 μm *z*-stacks located 2–4 μm apart were saved for analysis. Orthogonal views were created using ImageJ, and minimum adjustments were made to brightness and contrast in Adobe Photoshop.

### Statistics

Statistical analysis was done using Prism by GraphPad for Macintosh (Version 9). The specific test used for each experiment is notated in the figure legends. *P* values of < 0.05 were considered significant and are indicated as *P < 0.05; **P < 0.01; ***P < 0.001.

## SUPPLEMENTAL MATERIAL

Figure S1 spectrum.02595-24-s0001.pdf Supplemental data to support Figure 1

ASM does not own the copyrights to Supplemental Material that may be linked to, or accessed through, an article. The authors have granted ASM a non-exclusive, world-wide license to publish the Supplemental Material files. Please contact the corresponding author directly for reuse.
