# Supplementary material for: Listeria monocytogenes use multiple mechanisms to disseminate from the intestinal lamina propria to the mesenteric lymph nodes
Source: Microbiol Spectr. 2024 Dec 23;13(2):e02595-24. doi: 10.1128/spectrum.02595-24 (PMC11792513; doi:10.1128/spectrum.02595-24)
Supplement: Figure S1 — Supplemental data to support Figure 1 [file spectrum.02595-24-s0001.pdf]

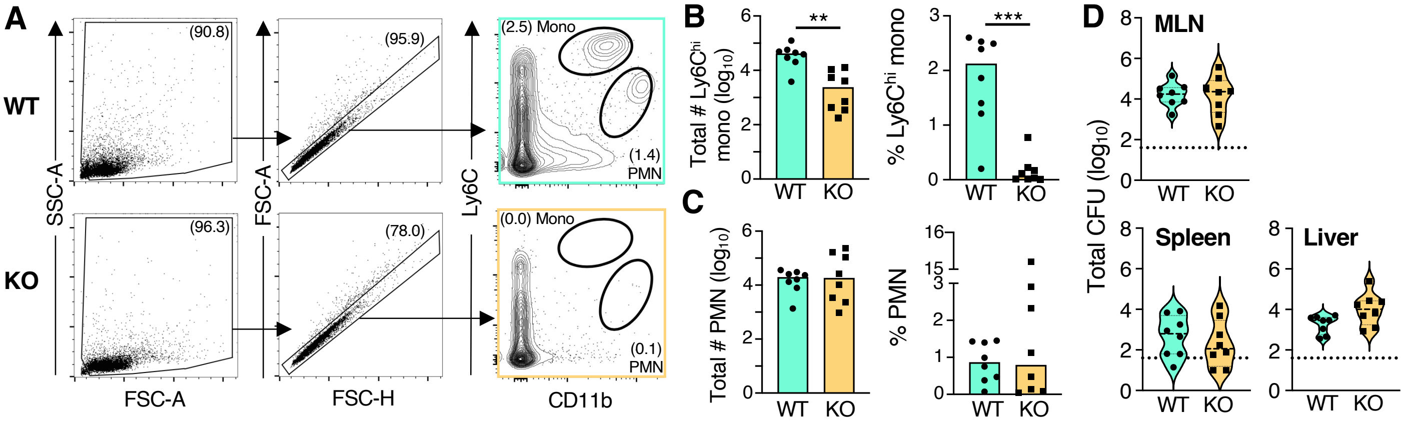

FIG S1 Supplemental Data to Support Figure 1. C57BL/6J (WT) and CCR2$^{-/-}$ (KO) mice were fed 3-4 x 10^8 CFU of *Lm* SD2001 and tissues were collected 2 dpi. Pooled data for n=8 mice analyzed in two independent experiments performed several years prior to experiments in Fig. 1 are shown. (**A**) Gating strategy for Ly6C^{hi} monocyte (Mono) and PMN populations in the pooled MLN of mice. (**B**) Median total number and percentage (of total live cells) of monocytes **(B)** and PMN **(C)** in the MLN as determined by flow cytometry. (**D**) Violin plots of the *Lm* SD2000 burden in the tissues 2 dpi; the dashed lines represent median, the dotted lines represent limits of detection, and the vertical segments represent interquartile range. Significance for all panels was calculated using Mann-Whitney test for unpaired data (**,$p < 0.05$; ***,$p < 0.001$).
